# Editorial: Elucidation of the causes of human disease by multi-omics integration

**DOI:** 10.3389/fgene.2023.1271406

**Published:** 2023-08-09

**Authors:** Marta Rusmini, Francesca Lantieri

**Affiliations:** ^1^ Laboratory of Genetics and Genomics of Rare Diseases, Giannina Gaslini Institute (IRCCS), Genova, Italy; ^2^ Department of Health Sciences, School of Medical and Pharmaceutical Sciences, University of Genoa, Genoa, Italy

**Keywords:** omic analyses, multi-omic, human diseases, bioinformatics, integration

This Research Topic collected papers aimed at integrating different omics data to investigate the causes of human diseases. The multi-omics analysis, in fact, can leverage the advantages of each set of data, collecting more complete biological and clinical information about patients and thus providing a more holistic point of view. Integrating analysis allows us to model the correlations or associations likely present at the different omics layers of related data leading to a better modeling of the complexity of data and the possibility of applying multivariate methods. Ultimately, these approaches can provide hints on the complex biological and molecular patterns underlying human traits and diseases. Several multi-omics approaches have been published in order to improve knowledge about human diseases, whether they are Mendelian diseases (Almeida et al., 2022), complex disorders (Chong et al., 2023; Diray-Arce et al., 2023) or tumors (Park et al., 2023; Poos et al., 2023).

One of the greatest advantages of integrating multi-omics data is the possibility to exploit different datasets already available. Thanks to the improvement in technologies and bioinformatic tools, as well as the more and more affordable costs, omics data of many kinds have been generated at a remarkable hastening. Alongside, many databases that include clinical data together with genomic data, expression profiles, and other omics data are progressively becoming available, on both specific diseases and general populations. Just think of widely used databasessuch as TCGA (https://www.cancer.gov/tcga) and GTEx database (https://gtexportal.org/home), and the newly released proteomic dataset by UK Biobank (https://www.ukbiobank.ac.uk/), just to name a few. Among the papers collected in this Research Topic, Chen et al. investigated the SHC-adaptor protein 1 (SHC1) role in cancer through a pan-cancer analysis taking advantage of data from public databases (GTEx database, TCGA database, Oncomine and CPTAC database). The SHC1 expression levels were examined with regard to several crucial issues related to cancer, such as clinical outcomes, genetic alteration, DNA methylation, or protein phosphorylation. Altogether, their results support the SHC1 role in tumor immunity and its possible prognostic and diagnostic value in multiple cancers. Also Xie et al. focused on cancer, taking advantage of the TCGA database. They used mRNA expression and clinical data of Pancreatic ductal adenocarcinoma (PDAC) patients from the TCGA database as training cohort to establish the prognostic model and from GEO database as test cohort to validate the model focusing on necroptosis-related genes (NRGs). They were thus able to construct a genetic risk prognostic model based on four NRGs, independent of other risk factors such as age and tumor grade and with a fair predictive diagnostic efficacy that might be used in PDAC patients.

Besides the articles described above, many studies based on multi-omics analysis are focused on the field of tumor and have the goal of identifying biomarkers with a diagnostic and prognostic effect. Kaya et al. combined a published dataset of whole genome gene expression from patients with colorectal cancer (CRC) with already published copy number alterations data of different CRC samples, in order to identify a gene signature with a potential diagnostic and predictive value. The authors evaluated this gene signature expression levels in blood samples from CRC patients using data from GTEx portal, to test blood as a biological sample from which to extract information in a non-invasive way. Also Chen et al. focused on CRC datasets. Through the development of a new method, they identified gene networks significantly correlated with CRC survival that included genes with crucial roles in cancer progression and that could serve as potential prognostic biomarkers. With the same purpose, Needhamnsen et al. published a study on patients affected by Multiple Sclerosis (MS) in which they combined small RNA data and methylome data by applying the multiple co-inertia analysis (MCIA) multivariate method. This unsupervised approach was applied to multi-omics data derived from multiple compartments, cell-free cerebrospinal fluid (CSF), CSF, plasma and peripheral blood mononuclear cells from the same 42 individuals. Their integrative approach allowed to distinguish between the relapsing-remitting form and the secondary progressive stage of MS patients.

An additional advantage of omics data is that they can be re-analyzed as information is added and new methods of analysis evolve. It is therefore compelling the development and improvement of analysis methods, their standardization and guidelines on both analysis and data control steps based on interdisciplinary approaches. Several articles provide an overview on the multi-omics analysis (Correa-Aguila et al., 2022) with particular regard to the machine learning approach (Reel et al., 2021). Athieniti and Spyrou also define the main objectives investigated through multi-omics and describe the different methods applied as well as the main challenges (Athieniti and Spyrou, 2023). In the present editorial, Chen et al., designed a framework, Correlation-based Local Approximation of Membership (CLAM), which combines multi-omics data to identify co-expressed gene modules without the need across different datasets to share the same genes or samples and exploiting known molecular interactions in module detection, thus overcoming some common limitations of current methods. They applied their method to expression data from several cancer and normal tissue dataset finding higher precision and biological relevance in modules retrieval and a higher number of gene ontology (GO) terms in enrichment analysis in comparison to other known methods.

Besides the necessity of improving data integration methods and guidelines, there are still several limitations to the multi-omics approach. Among the most evident, is the lack of external validation of the results obtained by multi-omics analysis in different biological samples. In addition, some of the common issues in human diseases studies are even more crucial in multi-omics studies. The risk of sampling bias and the importance of detailed phenotypes and clinical classification remains of utmost importance, including the definition of covariates related to clinical outcomes (Lipman et al., 2023). Finally, the sample size is of concern, since achieving the statistical power becomes even more relevant than in the single omics.

In conclusion, multi-omics analysis can improve the knowledge of the complex underlying biology and identify prognostic and diagnostic biomarkers, as attempted in the studies published in the present Research Topic. This might eventually pave the way to better prevention and personalized medicine: multi-omics are still at their dawn, with some methods still lacking maturity, but these approaches are definitely promising and worthy of being increasingly applied to the study of human traits and diseases.
